# O4-6 Citizen involvement and Active Living - a research project in the middle of Odense

**DOI:** 10.1093/eurpub/ckac094.030

**Published:** 2022-08-29

**Authors:** Danielle Nørager Johansen, Jens Troelsen, Thomas Kaarsted, Anne Kathrine Overgaard

**Affiliations:** 1 Department of Sports Science and Clinical Biomechanics, University of Southern Denmark, Odense, Denmark; 2 Citizen Science network, University of Southern Denmark, Odense, Denmark

**Keywords:** Citizen Science, Active Living, Citizen involvement, Health Enhancing Physical Activity

## Abstract

**Background:**

Physical inactivity and its consequences is a growing problem. Health enhancing physical activity (HEPA) behavior has previously been linked to the characteristics of the physical surroundings. Active Living (AL) is, in that connection, an international concept developed to integrate HEPA into everyday life.

The city of Odense located on Funen in Denmark has undergone major urban developments throughout the past 10 years - and will continue to do so prospectively. This has resulted in a possibility to create an AL area to be used by various target groups (employees and students at the university, patients and relative at the hospital, athletes participating in sporting activities in different associations etc.). Therefore, the University of Southern Denmark (SDU) is involved in a project aiming at creating a 80-hectare AL area with various activity possibilities for the everyday users.

**Methods:**

Citizen Science (CS) has been the bearing principle from the beginning of the project. Rather than developing such an area ‘behind the desk’, the guiding question throughout the process has been: According to the citizens, what should such area contain of? This has been initiated through expert workshops, student modules in relation to the Sports and Health education at SDU, arranging events together with local media, organizing the Danish Championship in cycle cross, organizing a school festival etc.

**Results:**

Using the CS approach, the project has received more than 1.000 inputs and ideas from citizens, associations, students etc. The proposals have formed the basis for the results in a combined PhD project, focusing on AL and principles of landscape architecture. Drawings from this PhD will be presented and principles of Citizen Science will be discussed at the conference.

The next project step is to raise external funding to realize the potential of the area.

**Conclusion:**

At this time, the project is still at its initial stage trying to raise funding to initiate and carry out the collected ideas. However, this project is a great example on how a CS approach in developing an urban area by integrating various sectors, hopefully, can improve the final product in terms of usability and sustainability.

